# Proteomic Analysis of Cerebrospinal Fluid From Patients With Extranodal NK-/T-Cell Lymphoma of Nasal-Type With Ethmoidal Sinus Metastasis

**DOI:** 10.3389/fonc.2019.01489

**Published:** 2020-01-10

**Authors:** Qingfang Li, Hao Zeng, Yunuo Zhao, Yanqiu Gong, Xuelei Ma

**Affiliations:** State Key Laboratory of Biotherapy, Department of Biotherapy, Cancer Center, West China Hospital, Sichuan University, Chengdu, China

**Keywords:** ENKTL, proteomics analysis, CSF, lymphoma, proteome

## Abstract

**Objective:** Extranodal natural killer /T-cell lymphoma (ENKTL) is an aggressive and unusual subtype of non-Hodgkin lymphoma (NHL) that it is related with the Epstein-Barr virus (EBV). CSF is considered as an ideal source of high-concenrtation disease-related proteins. We aimed at identifying the proteomic markers changes of CSF in ENKTL patients and used such changes to diagnose ENKTL.

**Materials and methods:** In this study, CSF samples were acquired from hospitalization patients from the Cancer Center of West China Hospital, Chengdu, China. Comparative proteomic profiling are commonly used to do label-free liquid chromatography-tandem mass spectrometry (LC-MS/MS). And in this study the same method was used to characterize the variety of proteins in ENKTL patients and none-ENKTL people.

**Results:** In the aggregate, 421 non-excrescent and functional proteins were identified among the samples. Of these proteins, 45 proteins quantified match the involved criteria. HRG, TIMP-1, SERPINA3, FGA, FGG, TF, FGB, APP, and AGT were significantly up-regulated.

**Discussion:** We discovered that some proteins were significantly up-regulated. Also, these proteins themselves or with others proteins may be potential markers to diagnose ENKTL. The changes of proteomics may be a potential method to precisely identify the pathogenesis of the ENKTL.

## Introduction

Extranodal natural killer/T-cell lymphoma (ENKTL) is an aggressive and unusual subtype of non-Hodgkin lymphoma (NHL) that is associated with the Epstein-Barr virus (EBV) (1, 2). The incidence of this disease is relatively high in East Asia and South America, while it is low in the Western world. The nasal cavity is the most frequently affected site, often involving the nasopharynx and non-nasal areas such as the skin, gastrointestinal tract, lung, liver, salivary gland, and testis (3). Fine needle aspiration or core needle biopsy is widely used in ENKTL diagnosis. Necrosis is often seen in diagnostic biopsies and this may result in a delay in diagnosis. In a study with a median follow-up of 44.9 months, 26% of all stage I or II patients and 71% of stage III or IV patients died (4). Concurrent chemoradiation, sequential chemoradiation, and sandwich chemoradiation are recommended to treat stage I or II patients by the National Comprehensive Cancer Network. Stage III or IV patients were suggested to undergo these treatments followed by radiotherapy (5–7). Disease progression was still the most important factor leading to death, while pathogenesis of ENKTL is still unknown. Recently, increasing numbers of biomarkers related to disease progression and prognosis have been described. The overexpression of enhancer of zeste homolog 2 (EZH2) and H3K27-specific histone methyltransferase in ENKTL patients' tissue is associated with disease progression and prognosis (8). The atypical cells found in the cerebrospinal fluid (CSF) of patients with ENKTL are positive for CD3, CD43, and CD56, and some studies use these markers to diagnose ENKTL (9, 10).

The CSF is formed primarily in the ventricular choroid plexus and exists within the ventricular system and subarachnoid space. The proteins in the CSF originate from three different sources: cells within the CSF, plasma, and neural tissue (11). The CSF is considered an ideal source of high-concentration, disease-related proteins and cells. The proteome of normal human CSF was detected using immunoaffinity depletion and 2D liquid chromatography tandem mass spectrometry with over 2,500 proteins being identified (12, 13). The proteome is widely used in the detection of protein in the CSF of patients. The CSF samples of patients with Alzheimer's disease were analyzed and the levels of 750 proteins changed, identifying four proteins that had levels different from those in healthy individuals (14). Approximately 19 proteins have been identified in eight peer-reviewed articles as proteins with differential expression proteins in the CSF of glioma patients (15). Biomarkers in the CSF to predict the conversion process from clinically isolated syndrome (CIS) to multiple sclerosis (MS) have been identified to reach a correct diagnosis (16).

To identify changes in proteomic markers of the CSF in ENKTL patients, we used label-free liquid chromatography-tandem mass spectrometry (LC-MS/MS) to process the samples. We found that histidine-rich glycoprotein (HRG), tissue inhibitor of matrix metalloproteinase-1 (TIMP-1), serpin family A member 3 (SERPINA3), fibrinogen alpha chain (FGA), fibrinogen gamma chain (FGG), transferrin (TF), fibrinogen beta chain (FGB), amyloid beta precursor protein (APP), and angiotensinogen (AGT) significantly differed between the two patient groups. These proteins may be potential markers of ENKTL and may be related to disease progression.

## Methods

### Patients

All enrolled ENKTL patients were diagnosed from 2016 to 2017. All participating patients or their guardians provided informed consent in accordance with a University of Sichuan Review Board-approved protocol. Assent was obtained when required. Patient demographics were obtained from medical records and included age at diagnosis, sex, ethnicity, and treatment protocol. Six individuals who were suspected of having meningitis with similar characteristics to those of the patients were recruited as the control group. Inclusion criteria comprised (1) age: >18 years, <70 years; (2) diagnosis of ENKTL; and (3) no history of cranial treatment. Exclusion criteria comprised (1) diagnosis of another tumor at the same time and (2) a history of previous nervous system disease.

### CSF and Plasma Sample Collections

To analyze the change in proteins in the CSF in ENKTL, the CSF was collected before the first cranial treatment. One milliliter of CSF was collected during therapeutic lumbar punctures. Samples were centrifuged at 3,000 rpm for 10 min to remove cellular debris, which was collected within 4 h of puncturing, and was stored in 1 mL containers at −80°C until performing the assay. Only samples that were clear and without visible blood were collected after centrifugation. The Medical Ethics Committee of West China Hospital, Sichuan University approved this study. All the involved patients provided signed informed consent in accordance with the Declaration of Helsinki.

### Chemicals and Materials

Acetonitrile (ACN, A955-1), water (W6-1), and formic acid (FA, A117-50) were all Optima LC/MS grade and obtained from Fisher Scientific for use in the LC/MS experiment. The desalting tips were ZipTip Pipette Tips (ZipTip C18, ZTC18S096, Merck Millipore). The water, which was bought from a Thermo Scientific Barnstead NANOpure Water Purification System, was applied in all experiments besides the LC-MS/MS sample preparation. Sequencing-grade modified trypsin (V5117) was acquired from Promega. Ammonium bicarbonate (NH4HCO3, 11213-1KG-R) was required from Sigma-Aldrich (St. Louis, MO, USA). Tryptic digestion was used in Reagents including DL-Dithiothreitol (V900830), iodoacetamide (IAM, l1149-5G), and L-cysteine (168149-100G) were obtained from Sigma-Aldrich (St. Louis, MO, USA).

### Trypsin Digestion

Protein in-solution digestion: Cold RIPA buffer with protease and phosphatase inhibitors was mixed with a fixed volume of CSF (30 μL) while on ice for 10 min. The RIPA buffer consisted of 50 mm Tris-HCl, pH 7.61; 150 mm NaCl; 1% deoxycholic acid; and NP-40. The samples were then centrifuged at 4°C at 20,000 *g* for 5 min. The Bradford protein assay was used to quantify the supernatant of the samples. To make an alkaline environment for the following trypsin digestion, 100 mM NH_4_HCO_3_ was used in the buffer that was mixed with the CSF. Protein samples were mixed with 5 mM DL-dithiothreitol for ~60 min at 37°C to reduce the disulfide bonds. Iodoacetamide was mixed at a final concentration of 15 mM and left to react in the dark for 45 min to alkylate the cysteines. Eventually, 30 mM L-cysteine was used to block surplus IAM. Trypsin (Sequencing Grade Modified Trypsin) from Promega was used to digest the CSF protein samples overnight at 37°C at a 50:1 protein to trypsin ratio and pH 8.0. CSF samples were heated to 90°C to deactivate the enzymes in order to stop digestion reactions. The desalted peptides, which were distributed with C18 ZipTips, were used in LC–MS/MS analysis.

### Mass Spectrometric Analysis

The MS analysis used a past method with slight modification (17). At the start of MS/MS, all involved CSF samples were lyophilized and resuspended in buffer A and LC-MS/MS analysis was executed using an LC instrument (EASY-nLC 1000, Thermo Fisher Scientific) nanoflow combined with a Q Exactive quadrupole-orbitrap mass spectrometer (Thermo Fisher Scientific). Magic C18 AQ resin (200 A, 5 μm; Michrom Bioresources) was used to pack a 100-μm × 2-cm trap column and a 75-μm × 12-cm analytical column. The mobile phases were consistent with A and B. A was composed of 2% ACN and 0.1% FA. A and B consisted of 95% ACN and 0.1% FA. The LC gradient elution arrived at 4% B for 180 s. After concentration, the samples reached 22% B from 180 s to 40 min, when the concentration was increased from 22 to 30% over the next 480 s. After B was at 90% from 52 min to 1 h, it was distributed at a flow rate of 300 nL/min for 300 s. The positive ion mode was used in acquiring dependent data. MS spectra from 350 m/z to 1,800 m/z were analyzed at a resolution of 7 × 10^4^ at m/z = 200. The automatic gain control was set at 3 × 10^6^ in advance, with peak fill times of 20 ms. During MS/MS scanning, the 20 strongest parent ions were picked using a 1.6 m/z insulation window and fragmented with a normalized collision energy of 27%. The automatic gain control target value of MS/MS was changed to 1 × 10^6^. The sample was distributed with a peak fill time of 64 ms and resolution of 1.75 × 10^4^. Whether the parent ions were in a *z* = 1 charged state or states with unassigned charge was ruled out based on fragmentation, with the intensity threshold set to 3.1 × 10^5^. Fragmentation was performed using an HCD collision cell with a mass resolution of 1.7 × 10^4^ at m/z = 200. An actional exclusion period of 30 s was employed with one count repeatedly.

### Data Analysis

The raw data obtained using the Q-Exactive plus were transferred to and analyzed using Maxquant v1.3. The studies were based on the SwissProt human database, which was updated in September 2017 with 20,239 sequences. To calculate the peptide false discovery rate (FDR), the studies were compared against the reverse dbase and were performed with a precursor peptide mass tolerance of 10 ppm. Two missed trypsin cleavages were identified in the study. Cysteine carbamidomethylation was determined as the settled revision. Oxidation of methionine and acetylation of protein N-terminals were identified as unstable revisions. Label-free quantification was performed using MaxQuant, following a previously described method (18). Peptides with an expectation value of <1% after false discovery rate (FDR) correction were selected for further data processing.

A comparative analysis of protein profiles of the ENKTL patients and control group was performed for candidate proteins that had a unique change using univariate analysis. The *P*-values were calculated based on the two-tailed *t*-test for parametric data and considered statistically significant when <0.05.

### Proteomic Analysis

In this study, we used David 6.8 (https://david.ncifcrf.gov/) to perform the enrichment analysis of the proteins for Gene Ontology (GO) terms and Kyoto Encyclopedia of Genes and Genomes (KEGG) pathways. The heat map of the global protein expression profiles was made using by R software. The interprotein interactions were identified using the String and cytoscope online with a combined score >0.4 considered significant (https://string-db.org/).

## Results

**Figure 1:**
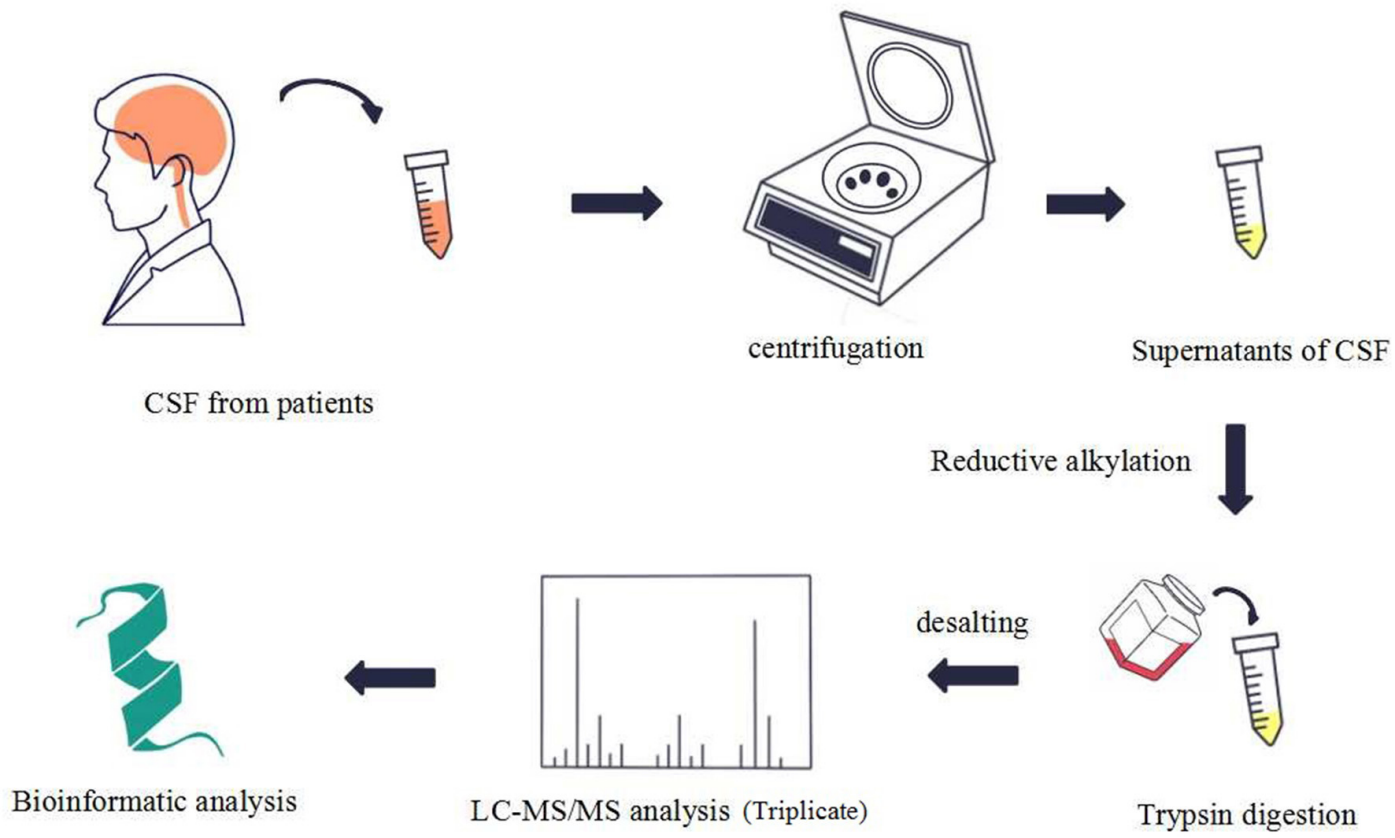
This figure shows the process of sample preparation, data acquisition, and analysis.

To identify the differences in the proteome between ENKTL patients and non-ENKTL controls, we collected CSF samples from 12 patients in West China hospital, six of whom had ENKTL and the rest were tumor-free patients suspected of having meningitis (patient information is listed in Supplementary Table 1). The CSF samples (Supplementary Table 2) were processed and analyzed using LC-MS/MS following the process outlined in Figure 1. We adopted a strict criterion to restrict the protein content to that based on the lowest special peptide after applying a 1% FDR standard according to the value of the peptide.

**Figure 2:**
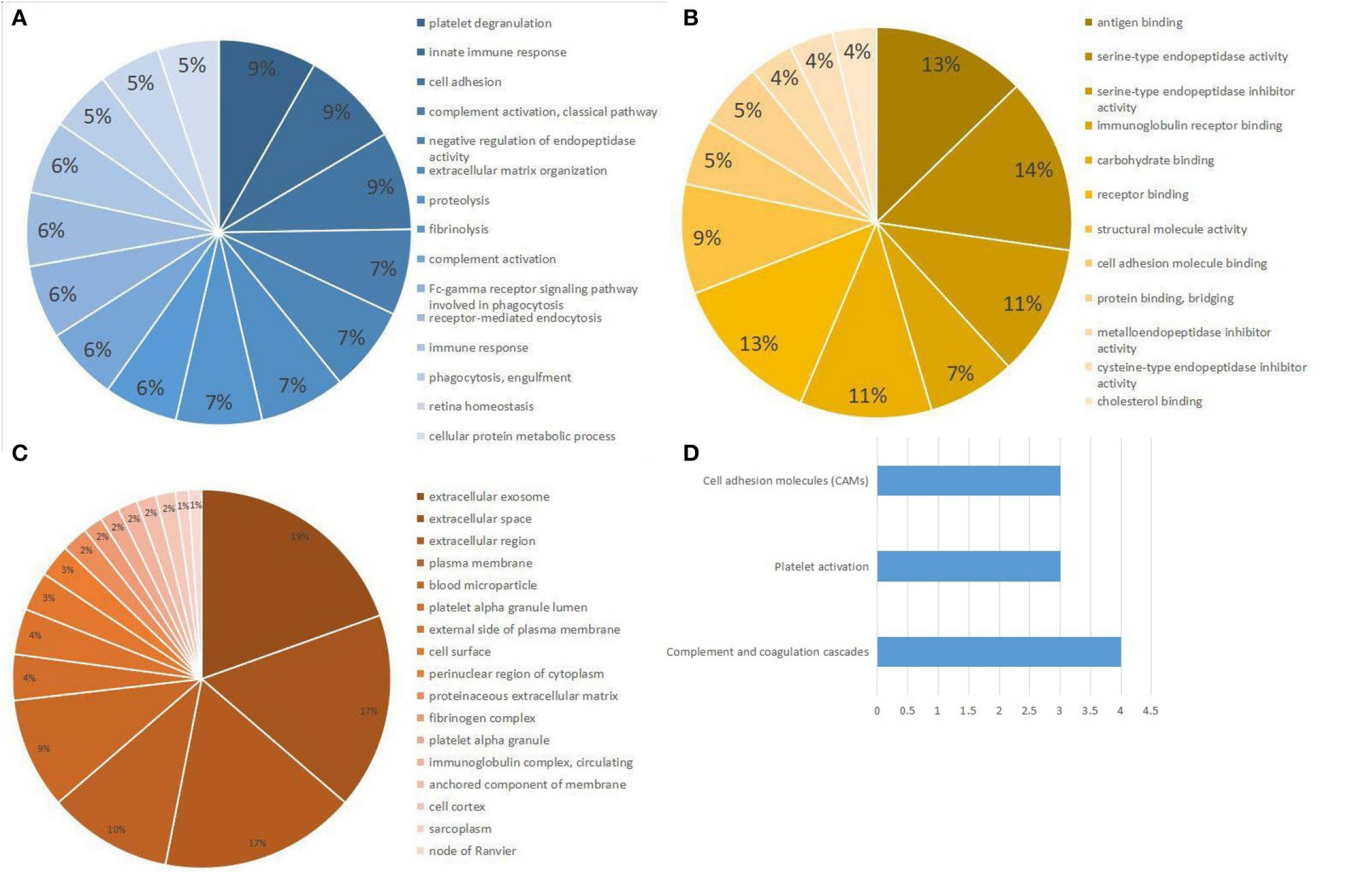
Gene Ontology (GO) and KEGG pathway analysis of differential expression proteins. **(A)** Biological processes obtained from GO analysis of all differential expression proteins. **(B)** Molecular function obtained from GO analysis of all differential expression proteins. **(C)** Cellular component analysis from GO analysis of all differential expression proteins. **(D)** Top three pathways obtained from KEGG pathway analysis of all differential expression proteins.

In the aggregate, 421 non-excrescent and functional proteins were identified among the samples. Of these proteins, 45 proteins were quantified matching the mentioned criteria. We then performed enrichment analysis for GO based on human proteins. We discovered that the response to platelet degranulation, innate immunity, cell adhesion, complement activation, negative regulation of endopeptidase activity, and extracellular matrix organization were significantly enriched (*P* < 0.001) in the biological procedural GO analysis (Figure 2A). The top seven, in terms of proportion of the quantified proteins in the GO molecular function analysis (Figure 2B), were antigen binding, serine-type endopeptidase activity, serine-type endopeptidase inhibitor activity, immunoglobin receptor binding, carbohydrate binding, receptor binding, and structural molecule activity. The cellular component analysis of proteins mainly indicated that they were highly correlated to the extracellular region (Figure 2C). The analysis of the KEGG-pathway indicated that the pathways containing complement and coagulation cascades, platelet activation, and cell adhesion molecules were enriched (Figure 2D).

**Figure 3:**
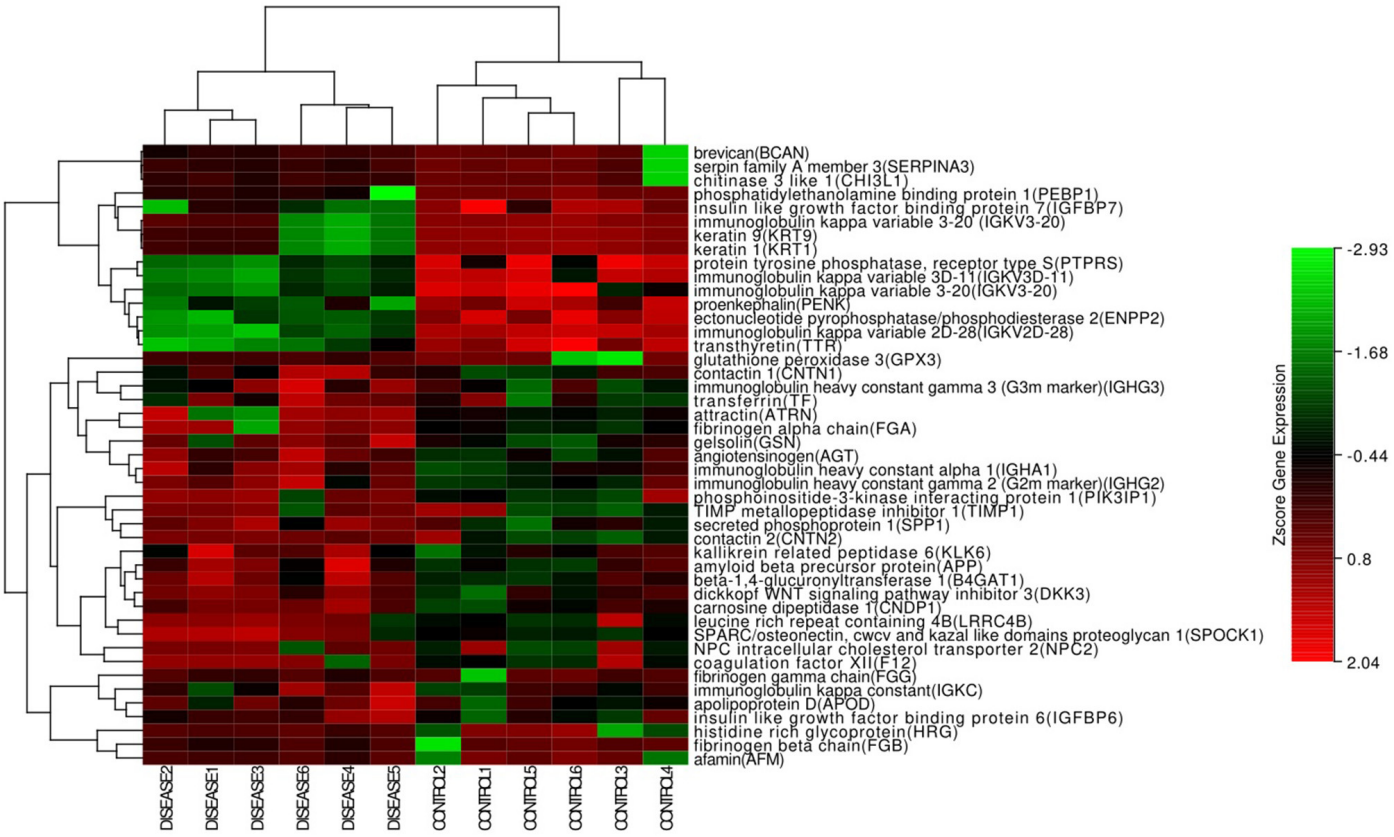
The hotmap of the proteins (Hierarchical clustering of global proteins).

**Figure 4:**
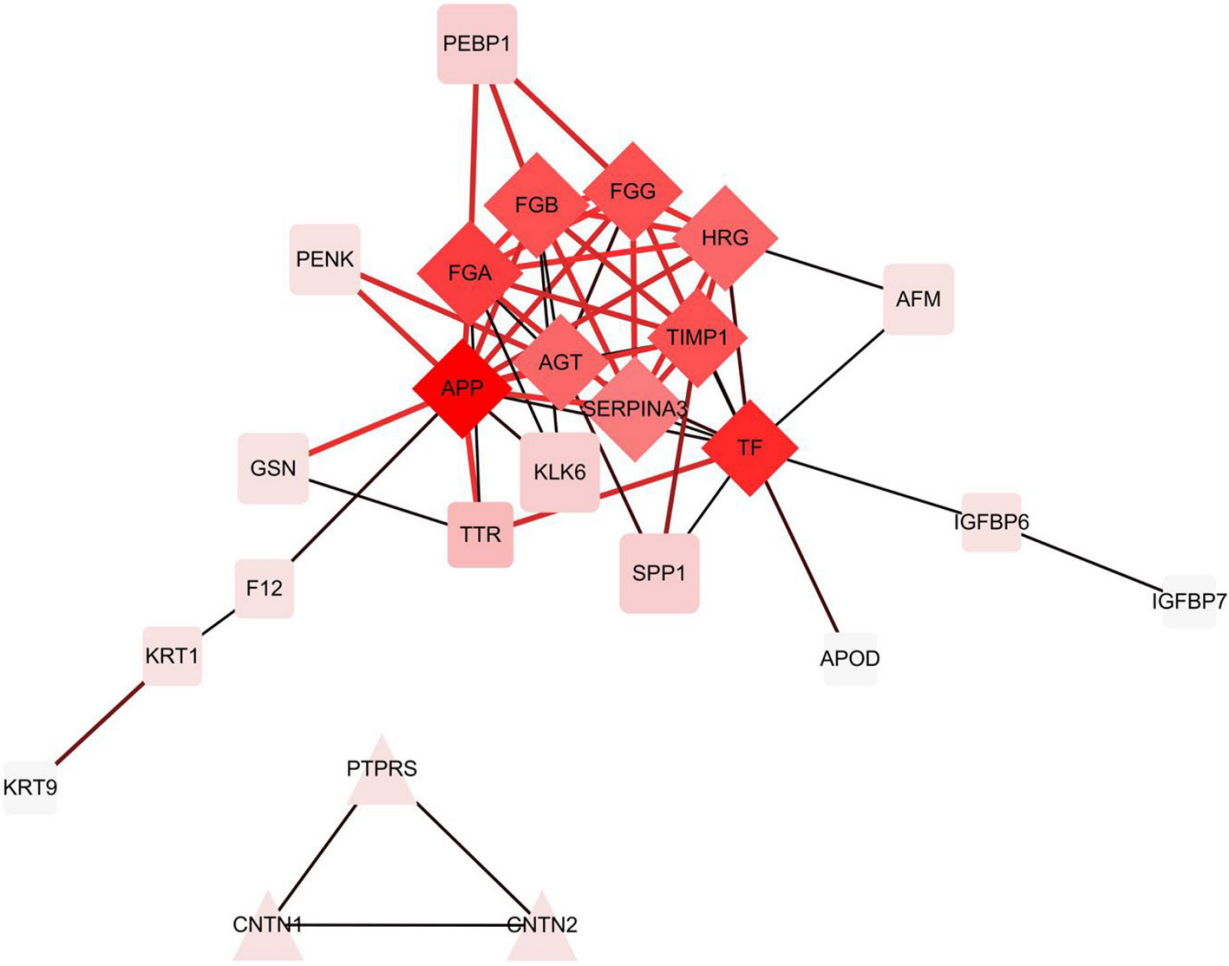
The differential expression proteins interactions analyzed by STRING [Protein -Protein Interaction (PPI) network].

In the hierarchical cluster analysis of the CSF proteome in ENKTL and control patients, only the differential expression proteins were taken into consideration (Figure 3). Twelve proteins were downregulated and 33 proteins were upregulated. We then used stings to analyze protein-to-protein interaction (Figure 4). We identified nine core proteins that were closely connected to each other and changed drastically between ENKTL and control patients. HRG, TIMP-1, SERPINA3, FGA, FGG, TF, FGB, APP, and AGT were all higher in ENKTL patients (Supplementary Figure 1).

## Discussion

To identify the differences between ENKTL patients and control patients, we performed LC-MS/MS analysis. In our study, we identified 421 proteins, of which 45 proteins were significantly different between groups. HRG, TIMP-1, SERPINA3, FGA, FGG, TF, FGB, APP, and AGT were significantly upregulated in ENKTL patients.

APP, A2M, and SERPINA 3 are acute phase proteins. APP is a single transmembrane protein expressed in a wide variety of cells and has a receptor-like structure. Several pathophysiological functions of APP have been discovered. APP is engaged in neuronal growth cone adhesion and plays an independent role as an operating cell adhesion molecule to bind to extracellular matrices (19). It was reported that the expression of APP is increased in both malignant breast cancer cell lines and breast cancer tissues (20). APP is also overexpressed in the papillary thyroid carcinoma and colorectal carcinoma (17, 21). Furthermore, the overexpression of APP is related to the prognosis. The function of A2M, which is a pan-proteinase inhibitors of the A2M family, is different from that of APP (22). A2M acts as a carrier protein and regulator for growth factors, polypeptide hormones, and cytokines. It is widely accepted that A2M is upregulated in human hepatocellular carcinoma (23). SERPINA3 is a protein of the serpin superfamily and is also known as α1-antichymotrypsin. SERPINA3 restricts the activation of several serine proteases, including chymotrypsin and cathepsin G. The overexpression of SERPINA3 leads to loss of adherence and delay of apoptosis (24). The upregulation of SERPINA3 has been reported in some cancer types, including endometrial carcinoma, lung carcinoma, colon carcinoma, breast carcinoma, and melanoma (25–30).

FGA, FGB, and FGG were all upregulated in the ENKTL patients. Two sets of α, β, and γ chains compose a large plasma protein called fibrinogen. These three chains are encoded by three independent genes. There are a number of reasons for fibrinogen upregulation, of which malignant tumor accounts for 20% based on a Chinese article. Previous studies indicate that the serum level of FGA is upregulated in many tumors, such as gastric cancer, breast cancer, nasopharyngeal carcinoma, acute lymphocytic leukemia, esophageal squamous cell carcinoma, and renal cell cancer (31–35). The abnormal expression of FGG was also reported in hepatocellular carcinoma and ovarian cancer (36). Furthermore, it was reported that the upregulation of FGB raised the expression of the other two. These three chains are interactively regulated in human cancer cells (37). The mechanism of the overexpression of these three proteins needs to be further explored.

TIMPs are specific inhibitors of matrixins and take part in controlling the local activities of matrix metalloproteinases (MMPs) in tissues (38–40). There are four known inhibitor subtypes referred to as TIMP-1, TIMP-2, TIMP-3, and TIMP-4. TIMP-1 inhibits MMP-9 with a high affinity. It has been proved that TIMPs promote cell growth and have anti-apoptotic activity (41). Disruption of the MMP-TIMP balance can lead to a number of pathogenic processes including tumor invasion, metastasis, and angiogenesis (42). According to a previous study, TIMP-1 levels are upregulated in non-small cell lung cancer and advanced breast carcinoma (43, 44). Additionally, it was reported that higher expression of TIMP-1 occurs in adenocarcinoma cells related to the higher stromal intensity of squamous cell carcinoma and non-small cell lung cancer (43). It was reported that the expression of TIMP-1 upregulated and promoted the development of EBV-associated malignancies in EBV-infected cells (45).

Previously, studies have shown that upregulation of SERPINA3 expression was an index of worse prognosis in ovarian cancer than low SERPINA3 level (46). A high TIMP-1 level was also associated with a worse prognosis (47, 48). The relationship of APP to prognosis are yet to be explored in tumors. In a further study, we will follow patients to observe the progression-free survival and overall survival and elucidate the relationship between proteins and survival.

There are some limitations of our study. First, the number of patients included in this research was limited. Second, there are various subtypes of NHL. ENKTL, an aggressive type of NHL, is special and difficult to distinguish. Therefore, more variable morphologic and immunophenotypic features are required when narrowing the differential diagnosis. The control group was also limited by the number of involved patients. In further studies, more control groups can be used, including diffuse large B cell lymphoma, Burkitt's lymphoma, or other types of lymphoma.

## Conclusion

We performed a proteomic analysis of the CSF in ENKTL patients, using a label-free method to compare proteomic marker variation between tumor patients and non-tumor patients. We discovered that HRG, TIMP-1, SERPINA3, FGA, FGG, TF, FGB, APP, and AGT were significantly upregulated and are potential markers for diagnosing ENKTL, either individually or in combination with each other. It is possible that these proteins play an important role in the occurrence of ENKTL lymphoma. The proteomic changes are a potential method of precisely identifying the pathogenesis of ENKTL.

## Data Availability Statement

The raw data supporting the conclusions of this manuscript will be made available by the authors, without undue reservation, to any qualified researcher.

## Ethics Statement

This study was approved by Review Board of Sichuan University. Patients Informed Consent was obtained from all participating patients or guardians in accordance with a University of Sichuan Review Board-approved protocol. This study was carried out in accordance with the recommendations of Sichuan University with written informed consent from all subjects. All subjects gave written informed consent in accordance with the Declaration of Helsinki. The protocol was approved by the Sichuan University.

## Author Contributions

QL and XM mainly generated with the idea and collected the data. QL wrote the manuscript. HZ and YZ helped to correct the grammar and does some statistical work. YG did the trials.

### Conflict of Interest

The authors declare that the research was conducted in the absence of any commercial or financial relationships that could be construed as a potential conflict of interest.

## References

[1] Su YJ Wang PN Chang H Shih LY Lin TL Kuo MC. Extranodal NK/T-cell lymphoma, nasal type: clinical features, outcome, and prognostic factors in 101 cases. Eur J Haematol. (2018) 101:379–88. 10.1111/ejh.13126 29908084

[2] Yamaguchi M Oguchi M Suzuki R. Extranodal NK/T-cell lymphoma: Updates in biology and management strategies. Best Pract Res Clin Haematol. (2018) 31:315–21. 10.1016/j.beha.2018.07.002 30213402

[3] Tse E Kwong YL. Diagnosis and management of extranodal NK/T cell lymphoma nasal type. Expert Rev Hematol. (2016) 9:861–71. 10.1080/17474086.2016.1206465 27347812

[4] Kim SJ Yoon DH Jaccard A Chng WJ Lim ST Hong H. A prognostic index for natural killer cell lymphoma after non-anthracycline-based treatment: a multicentre, retrospective analysis. Lancet Oncol. (2016) 17:389–400. 10.1016/S1470-2045(15)00533-1 26873565

[5] Yamaguchi M Tobinai K Oguchi M Ishizuka N Kobayashi Y Isobe Y. Concurrent chemoradiotherapy for localized nasal natural killer/T-cell lymphoma: an updated analysis of the Japan clinical oncology group study JCOG0211. J Clin Oncol. (2012) 30:4044–6. 10.1200/JCO.2012.45.6541 23045573

[6] Lee J Kim CY Park YJ Lee NK. Sequential chemotherapy followed by radiotherapy versus concurrent chemoradiotherapy in patients with stage I/II extranodal natural killer/T-cell lymphoma, nasal type. Blood Res. (2013) 48:274–81. 10.5045/br.2013.48.4.274 24466552 PMC3894386

[7] Wang L Wang ZH Chen XQ Wang KF Huang HQ Xia ZJ. First-line combination of GELOX followed by radiation therapy for patients with stage IE/IIE ENKTL: an updated analysis with long-term follow-up. Oncol Lett. (2015) 10:1036–40. 10.3892/ol.2015.3327 26622621 PMC4509369

[8] Liu J Liang L Huang S Nong L Li D Zhang B. Aberrant differential expression of EZH2 and H3K27me3 in extranodal NK/T-cell lymphoma, nasal type, is associated with disease progression and prognosis. Hum Pathol. (2019) 83:166–76. 10.1016/j.humpath.2018.08.025 30218753

[9] Walavalkar V Oak J Gu M. Cytological diagnosis of extranodal NK/T-cell lymphoma, nasal type, in cerebrospinal fluid. Cytopathology. (2013) 24:342–4. 10.1111/j.1365-2303.2012.00999.x 22844965

[10] Dunning KK Wudhikarn K Safo AO Holman CJ McKenna RW Pambuccian SE. Adrenal extranodal NK/T-cell lymphoma diagnosed by fine-needle aspiration and cerebrospinal fluid cytology and immunophenotyping: a case report. Diagn Cytopathol. (2009) 37:686–95. 10.1002/dc.21077 19373919

[11] Reiber H. Proteins in cerebrospinal fluid and blood: barriers, CSF flow rate and source-related dynamics. Restor Neurol Neurosci. (2003) 21:79–96. 14530572

[12] Schutzer SE Liu T Natelson BH Angel TE Schepmoes AA Purvine SO. Establishing the proteome of normal human cerebrospinal fluid. PLoS ONE. (2010) 5:e10980. 10.1371/journal.pone.0010980 20552007 PMC2881861

[13] Guldbrandsen A Vethe H Farag Y Oveland E Garberg H Berle M. In-depth characterization of the cerebrospinal fluid (CSF) proteome displayed through the CSF proteome resource (CSF-PR). Mol Cell Proteomics. (2014) 13:3152–63. 10.1074/mcp.M114.038554 25038066 PMC4223498

[14] Dayon L Núñez Galindo A Wojcik J Cominetti O Corthésy J Oikonomidi A. Alzheimer disease pathology and the cerebrospinal fluid proteome. Alzheimers Res Ther. (2018) 10:66. 10.1186/s13195-018-0397-4 30021611 PMC6052524

[15] Shen F Zhang Y Yao Y Hua W Zhang HS Wu JS. Proteomic analysis of cerebrospinal fluid: toward the identification of biomarkers for gliomas. Neurosurg Rev. (2014) 37:367–80; discussion: 380. 10.1007/s10143-014-0539-5 24781189

[16] Timirci-Kahraman O Karaaslan Z Tuzun E Kurtuncu M Baykal AT Gunduz T. Identification of candidate biomarkers in converting and non-converting clinically isolated syndrome by proteomics analysis of cerebrospinal fluid. Acta Neurol Belg. (2018) 119:101–111. 10.1007/s13760-018-0954-4 29873030

[17] Takayama K Tsutsumi S Suzuki T Horie-Inoue K Ikeda K Kaneshiro K. Amyloid precursor protein is a primary androgen target gene that promotes prostate cancer growth. Cancer Res. (2009) 69:137–42. 10.1158/0008-5472.CAN-08-3633 19117996

[18] Rardin MJ Newman JC Held JM Cusack MP Sorensen DJ Li B. Label-free quantitative proteomics of the lysine acetylome in mitochondria identifies substrates of SIRT3 in metabolic pathways. Proc Natl Acad Sci USA. (2013) 110:6601–6. 10.1073/pnas.1302961110 23576753 PMC3631688

[19] Young-Pearse TL Bai J Chang R Zheng JB LoTurco JJ Selkoe DJ. A critical function for beta-amyloid precursor protein in neuronal migration revealed by *in utero* RNA interference. J Neurosci. (2007) 27:14459–69. 10.1523/JNEUROSCI.4701-07.2007 18160654 PMC6673432

[20] Lim S Yoo BK Kim HS Gilmore HL Lee Y Lee HP. Amyloid-β precursor protein promotes cell proliferation and motility of advanced breast cancer. BMC Cancer. (2014) 14:928. 10.1186/1471-2407-14-928 25491510 PMC4295427

[21] Yang Z Fan Y Deng Z Wu B Zheng Q. Amyloid precursor protein as a potential marker of malignancy and prognosis in papillary thyroid carcinoma. Oncol Lett. (2012) 3:1227–30. 10.3892/ol.2012.639 22783423 PMC3392581

[22] Barrett AJ Starkey PM. The interaction of alpha 2-macroglobulin with proteinases. Characteristics and specificity of the reaction, and a hypothesis concerning its molecular mechanism. Biochem J. (1973) 133:709–24. 10.1042/bj1330709 4201304 PMC1177761

[23] Kar P Gandhi BM Irshad M Gupta H Tandon BN. Alpha-2 macroglobulin: an additional marker for diagnosis of hepatocellular carcinoma. J Assoc Phys India. (1987) 35:288–9. 2443479

[24] Chelbi ST Wilson ML Veillard AC Ingles SA Zhang J Mondon F. Genetic and epigenetic mechanisms collaborate to control SERPINA3 expression and its association with placental diseases. Hum Mol Genet. (2012) 21:1968–78. 10.1093/hmg/dds006 22246292

[25] Zhou J Cheng Y Tang L Martinka M Kalia S. Up-regulation of SERPINA3 correlates with high mortality of melanoma patients and increased migration and invasion of cancer cells. Oncotarget. (2017) 8:18712–25. 10.18632/oncotarget.9409 27213583 PMC5386641

[26] Yang GD Yang XM Lu H Ren Y Ma MZ Zhu LY. SERPINA3 promotes endometrial cancer cells growth by regulating G2/M cell cycle checkpoint and apoptosis. Int J Clin Exp Pathol. (2014) 7:1348–58. 24817931 PMC4014215

[27] Jin Y Wang J Ye X Su Y Yu G Yang Q. Identification of GlcNAcylated alpha-1-antichymotrypsin as an early biomarker in human non-small-cell lung cancer by quantitative proteomic analysis with two lectins. Br J Cancer. (2016) 114:532–44. 10.1038/bjc.2015.348 26908325 PMC4782198

[28] Karashima S Kataoka H Itoh H Maruyama R Koono M. Prognostic significance of alpha-1-antitrypsin in early stage of colorectal carcinomas. Int J Cancer. (1990) 45:244–50. 10.1002/ijc.2910450207 2303291

[29] Yamamura J Miyoshi Y Tamaki Y Taguchi T Iwao K Monden M. mRNA expression level of estrogen-inducible gene, alpha 1-antichymotrypsin, is a predictor of early tumor recurrence in patients with invasive breast cancers. Cancer Sci. (2004) 95:887–92. 10.1111/j.1349-7006.2004.tb02198.x 15546506 PMC11159604

[30] Montel V Pestonjamasp K Mose E Tarin D. Tumor-host interactions contribute to the elevated expression level of alpha1-antichymotrypsin in metastatic breast tumor xenografts. Differentiation. (2005) 73:88–98. 10.1111/j.1432-0436.2005.07302001.x 15811132

[31] Liu C Pan C Liang Y. Screening and identification of serum proteomic biomarkers for gastric adenocarcinoma. Exp Ther Med. (2012) 3:1005–9. 10.3892/etm.2012.515 22970007 PMC3438544

[32] Tao YL Li Y Gao J Liu ZG Tu ZW Li G. Identifying FGA peptides as nasopharyngeal carcinoma-associated biomarkers by magnetic beads. J Cell Biochem. (2012) 113:2268–78. 10.1002/jcb.24097 22334501

[33] Bai J He A Huang C Yang J Zhang W Wang J. Serum peptidome based biomarkers searching for monitoring minimal residual disease in adult acute lymphocytic leukemia. Proteome Sci. (2014) 12:49. 10.1186/s12953-014-0049-y 25317080 PMC4195909

[34] Wu C Luo Z Tang D Liu L Yao D Zhu L. Identification of carboxyl terminal peptide of Fibrinogen as a potential serum biomarker for gastric cancer. Tumour Biol. (2016) 37:6963–70. 10.1007/s13277-015-4394-y 26662807

[35] Yang J Xiong X Liu S Zhu J Luo M Liu L. Identification of novel serum peptides biomarkers for female breast cancer patients in Western China. Proteomics. (2016) 16:925–34. 10.1002/pmic.201500321 26705257

[36] Zhu WL Fan BL Liu DL Zhu WX. Abnormal expression of fibrinogen gamma (FGG) and plasma level of fibrinogen in patients with hepatocellular carcinoma. Anticancer Res. (2009) 29:2531–4. 19596924

[37] Roy SN Mukhopadhyay G Redman CM. Regulation of fibrinogen assembly. Transfection of Hep G2 cells with B beta cDNA specifically enhances synthesis of the three component chains of fibrinogen. J Biol Chem. (1990) 265:6389–93. 2318859

[38] Sternlicht MD Werb Z. How matrix metalloproteinases regulate cell behavior. Annu Rev Cell Dev Biol. (2001) 17:463–516. 10.1146/annurev.cellbio.17.1.463 11687497 PMC2792593

[39] Woessner JF. Role of matrix proteases in processing enamel proteins. Connect Tissue Res. (1998) 39:69–73; discussion: 141–9. 10.3109/03008209809023913 11062989

[40] Huppertz B Kertschanska S Demir AY Frank HG Kaufmann P. Immunohistochemistry of matrix metalloproteinases (MMP), their substrates, and their inhibitors (TIMP) during trophoblast invasion in the human placenta. Cell Tissue Res. (1998) 291:133–48. 10.1007/s004410050987 9394051

[41] Jiang Y Goldberg ID Shi YE. Complex roles of tissue inhibitors of metalloproteinases in cancer. Oncogene. (2002) 21:2245–52. 10.1038/sj.onc.1205291 11948407

[42] Salimi Sartakhti J Manshaei MH Sadeghi M. MMP-TIMP interactions in cancer invasion: an evolutionary game-theoretical framework. J Theor Biol. (2017) 412:17–26. 10.1016/j.jtbi.2016.09.019 27670802

[43] An HJ Lee YJ Hong SA Kim JO Lee KY Kim YK. The prognostic role of tissue and serum MMP-1 and TIMP-1 expression in patients with non-small cell lung cancer. Pathol Res Pract. (2016) 212:357–64. 10.1016/j.prp.2015.11.014 26995105

[44] Ławicki S Zajkowska M Głazewska EK Bedkowska GE Szmitkowski M. Plasma levels and diagnostic utility of VEGF, MMP-9, and TIMP-1 in the diagnosis of patients with breast cancer. Onco Targets Ther. (2016) 9:911–9. 10.2147/OTT.S99959 26966379 PMC4771393

[45] Lin SJ Wu SW Chou YC Lin JH Huang YC Chen MR. Novel expression and regulation of TIMP-1 in Epstein Barr virus-infected cells and its impact on cell survival. Virology. (2015) 481:24–33. 10.1016/j.virol.2015.02.015 25765004

[46] Jinawath N Vasoontara C Jinawath A Fang X Zhao K Yap KL. Oncoproteomic analysis reveals co-upregulation of RELA and STAT5 in carboplatin resistant ovarian carcinoma. PLoS ONE. (2010) 5:e11198. 10.1371/journal.pone.0011198 20585448 PMC2887843

[47] Chirco R Liu XW Jung KK Kim HR. Novel functions of TIMPs in cell signaling. Cancer Metastasis Rev. (2006) 25:99–113. 10.1007/s10555-006-7893-x 16680576

[48] Egeblad M Werb Z. New functions for the matrix metalloproteinases in cancer progression. Nat Rev Cancer. (2002) 2:161–74. 10.1038/nrc745 11990853

