# Supplementary material for: Proteomic Analysis of Cerebrospinal Fluid From Patients With Extranodal NK-/T-Cell Lymphoma of Nasal-Type With Ethmoidal Sinus Metastasis
Source: Front Oncol. 2020 Jan 10;9:1489. doi: 10.3389/fonc.2019.01489 (PMC6966716; doi:10.3389/fonc.2019.01489)
Supplement: Supplementary file 1 [file Table_1.DOCX]

Supplementary Table 1:Information of the CSF.

| patient number | 1 | 2 | | 3 | 4 | 5 | 6 |
| --- | --- | --- | --- | --- | --- | --- | --- |
| Appearance | colorless transparent | | colorless transparent | colorless transparent | colorless transparent | colorless transparent | colorless transparent |
| Pressure(mmHg) | 73 | | 160 | 88 | 80 | unavailable | 76 |
| nucleated cells count(10^6) | 0 | | 10 | 0 | 0 | 0 | 10 |
| Red cells count(10^6) | 0 | | 220 | 0 | 0 | 0 | 360 |
| Glucose(mmol/L) | 6.38 | | 4.34 | 2.83 | 2.46 | 3.65 | 3.98 |
| Chlorine(mmol/L) | 125.7 | | 127.6 | 117.5 | 123.4 | 130 | 122.9 |
| Protein(g/L) | 0.28 | | 0.44 | 0.27 | 0.23 | 0.43 | 0.79 |

Information of the CSF
