# Supplementary material for: Proteomic Analysis of Cerebrospinal Fluid From Patients With Extranodal NK-/T-Cell Lymphoma of Nasal-Type With Ethmoidal Sinus Metastasis
Source: Front Oncol. 2020 Jan 10;9:1489. doi: 10.3389/fonc.2019.01489 (PMC6966716; doi:10.3389/fonc.2019.01489)
Supplement: Supplementary file 2 [file Table_2.DOCX]

Supplementary Table 2:Basic characteristics of the patients and blood tests.

| patient number | 1 | 2 | 3 | 4 | 5 | 6 |
| --- | --- | --- | --- | --- | --- | --- |
| Sex | female | female | female | male | male | male |
| Age (year) | 41 | 49 | 43 | 58 | 38 | 48 |
| B symptoms | Present | Present | Present | Absent | Present | Present |
| Ann Arbor stage | IEB | IEB | IEB | IV | IVEB | IIEB |
| ECOG score standard | 0 | 0 | 0 | 1 | 1 | 0 |
| International Prognostic Index(IPI) | 1 | 2 | 2 | 2 | 4 | 3 |
| Distant metastasis | No | No | No | Yes | Yes | No |
| Chemotherapy regimens | VDLP | VDLP | VDLP | VDLP | VDLP | VDLP |
| Radiotherapy | Yes | Yes | Yes | Yes | Yes | Yes |
| LDH elevation | No | No | No | No | Yes | Yes |
| EBER1/2 FISH | + | + | + | + | + | + |
| Nasal Operation History | cut the polyp two months ago | Underwent augmentation rhinoplasty in 1997 | - | - | - | - |
| Red cell count(10^12/L) | 5.77 | 4.27 | 4.73 | 4.17 | 4.15 | 5.05 |
| Hemoglobin(g/L) | 109 | 123 | 114 | 112 | 121 | 150 |
| Platelet count(10^9/L) | 210 | 195 | 159 | 234 | 186 | 167 |
| White cell count(10^9/L) | 6.98 | 3.39 | 3.52 | 4.27 | 3.84 | 3.38 |
