# Supplementary material for: Proteomic Analysis of Cerebrospinal Fluid From Patients With Extranodal NK-/T-Cell Lymphoma of Nasal-Type With Ethmoidal Sinus Metastasis
Source: Front Oncol. 2020 Jan 10;9:1489. doi: 10.3389/fonc.2019.01489 (PMC6966716; doi:10.3389/fonc.2019.01489)
Supplement: Supplementary file 3 [file Presentation_1.PPTX]

## Slide 1
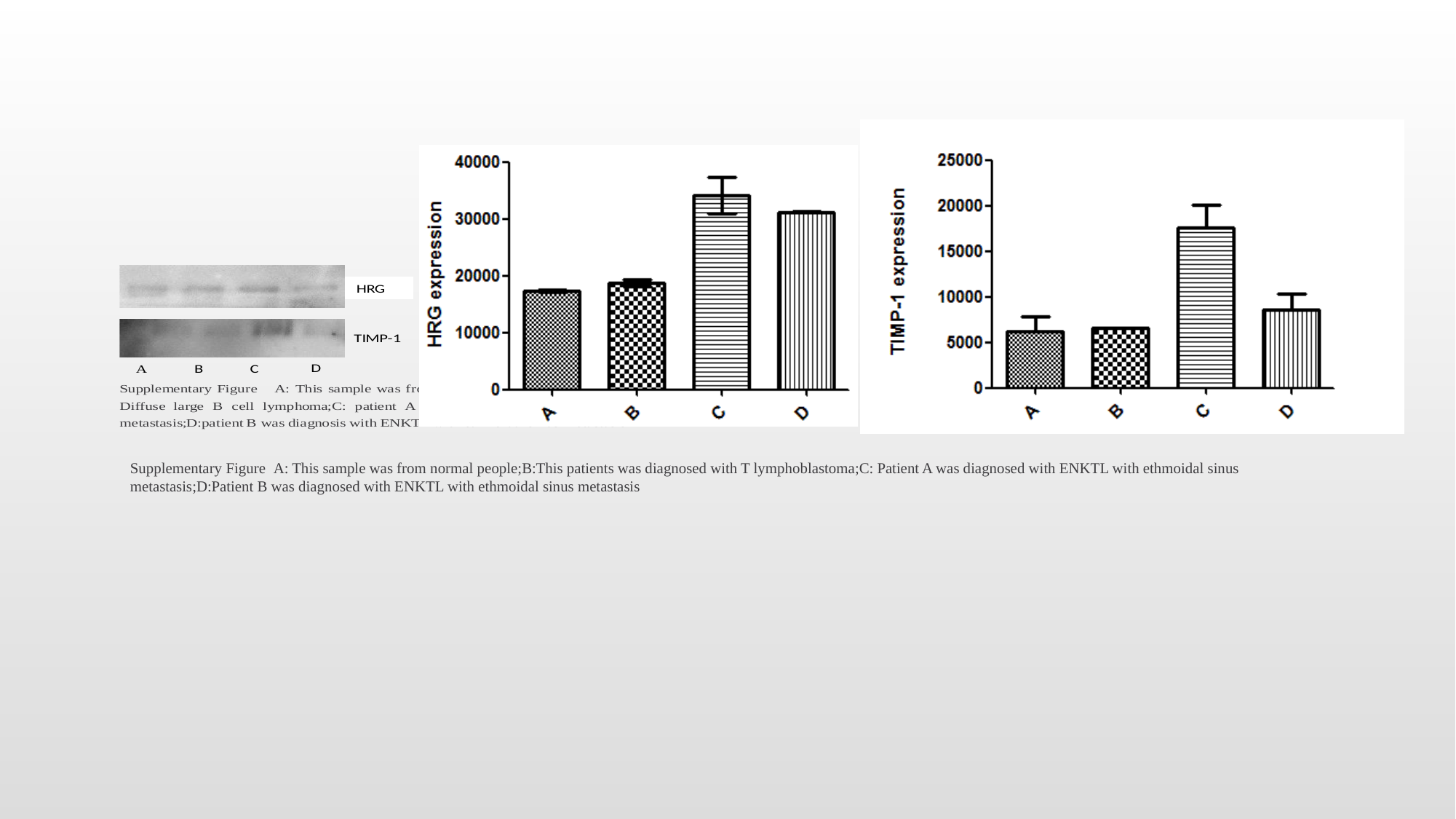

Supplementary Figure A: This sample was from normal people;B:This patients was diagnosed with T lymphoblastoma;C: Patient A was diagnosed with ENKTL with ethmoidal sinus metastasis;D:Patient B was diagnosed with ENKTL with ethmoidal sinus metastasis
